# PpSKα boosts chilling tolerance by activating PpTrxh9 in peach fruit

**DOI:** 10.3389/fpls.2025.1603423

**Published:** 2025-06-13

**Authors:** Caifeng Jiao, Jing Sun

**Affiliations:** ^1^ School of Horticulture, Anhui Agricultural University, Hefei, China; ^2^ College of Food Science and Engineering, Nanjing University of Finance and Economics, Nanjing, China

**Keywords:** chilling tolerance, SHAGGY-related protein kinase α, thioredoxin h9, oxidative damage, peach fruit

## Abstract

**Introduction:**

The cold chain system is extensively used to lower the quality deterioration in postharvest fruits. However, the occurrence of chilling injury (CI) is a common phenomenon in peach fruits, consequently compromising their market value.

**Methods and results:**

Cold tolerance was boosted by glycine betaine (GB) supplementation. GB treatment promoted the thioredoxin h9 (PpTrxh9) expression and ameliorated oxidative injury. Using assays such as yeast two-hybrid, co-immunoprecipitation, pull-down, and bimolecular fluorescence complementation, SHAGGY-related protein kinase a (PpSKa) was verified as a protein interacting with PpTrxh9. GB treatment elevated the PpSKa expression. The overexpression of PpSKa in tomato fruits decreased the cold sensitivity and oxidative damage, whereas virus-induced gene silencing of PpSKa in peach fruits aggravated the CI progression and oxidative damage. PpSKa was found to phosphorylate PpTrxh9 in the kinase assay. Moreover, PpSKa-overexpressed tomato fruits exhibited higher SlTrxh9 expression, whereas the PpSKa-RNAi peach fruit exhibited lower PpTrxh9 expression.

**Discussion:**

Taken together, PpSKa decreased the development of CI by improving the expression of PpTrxh9 in peach fruits.

## Introduction

Cold storage is conducive to retarding the decline in quality in postharvest fruits (Chen et al., 2022). However, chilling injury (CI) easily occurs in peach fruits along with refrigerated storage under low temperatures (Hu et al., 2022). Chilled peaches often exhibit internal browning (Jia et al., 2023). This damage reduces the commodity value of peach fruits. Glycine betaine (GB) supplementation has been reported to lower the sensitivity to cold stress during prolonged storage of peach fruits (Jia et al., 2022; Shan et al., 2016; Wang et al., 2019). Little is known regarding the downstream signal transduction pathways involved in the GB-reduced CI progression in peaches.

The occurrence of oxidative damage under cold stress as a consequence of the burst of reactive oxygen species (ROS) gives rise to the development of CI in postharvest fruits (Zhang et al., 2021). Amelioration of the oxidative damage would contribute to rendering the fruits more tolerant to cold stress. The application of GB was found to inhibit the membrane lipid peroxidation in cold-stored peach fruits (Wang et al., 2019). More physiological mechanisms of the suppression of oxidative damage with GB treatment need to be revealed. Thioredoxins (Trxs), as small ubiquitous disulfide reductases, play critical roles in reducing ROS overproduction, thereby maintaining the redox balance in plant stress adaptations (Sevilla et al., 2023). Plant Trxs are mainly divided into seven groups according to their subcellular localization: PpTrxf, PpTrxh, PpTrxm, PpTrxo, PpTrxx, PpTrxy, and PpTrxz (Fonseca-Pereira et al., 2021). However, the regulation of PpTrxs by GB supplementation in postharvest peach fruits under cold stress needs to be investigated.

It has been reported that the phosphorylation of target proteins by diverse protein kinases is involved in the defense against cold stress (Ding et al., 2019). Glycogen synthase kinase 3 (GSK3) members are highly conserved serine/threonine protein kinases. A larger family of GSK3/SHAGGY-like kinases (SKs) exists in plants (Youn and Kim, 2015). The impacts of GSK3/SKs on controlling multiple responses to environmental factors have been largely shown in various plant species (Li et al., 2021). In our earlier work, the addition of bikinin (a GSK3/SK inhibitor) decreased the chilling tolerance during cold storage in peach fruits, indicating that GSK3/SKs may be a positive regulator of chilling tolerance (Jiao and Duan, 2019a, 2019). However, the molecular mechanisms of the modulation of the responses to cold stress by GSK3/SKs in cold-stored peach fruits are largely unknown. Moreover, GSK3/SKs affect the activity and stability of downstream proteins via physical interactions and phosphorylation reactions, thus modulating the diverse defense responses. BIN2 in *Arabidopsis* interacted with and phosphorylated the inducer of CBF expression 1 (ICE1), thus modulating its level in response to low-temperature stress (Ye et al., 2019). ASKα from *Arabidopsis* phosphorylated and upregulated glucose-6-phosphate dehydrogenase, contributing to antioxidant defense (Dal Santo et al., 2012). More notably, the GSK3-like kinase BRASSINOSTEROID-INSENSITIVE2 (BIN2) in *Arabidopsis* was reported to alter the hydrogen peroxide level (Lu et al., 2022). *GmBIN2* from soybean contributed to lowering the oxidative damage and thus resisted salt and drought stress (Wang et al., 2018). ASKα from *Arabidopsis* facilitated scavenging of ROS production, thereby promoting resistance to salt stress (Dal Santo et al., 2012). *MmSK* from mulberry alleviated oxidative injury, therefore reducing the sensitivity to drought stress (Li et al., 2018). These reports implicated the function of plant GSK3/SKs in maintaining redox equilibrium. Thus, both PpSKs and PpTrxs can engage in redox equilibrium. The regulation of PpTrxs by PpSKs via protein–protein interaction and phosphorylation in order to inhibit oxidative damage in cold-stored peaches remains to be substantiated.

In this research, the modulation of CI progression and PpTrxh9 expression by GB supplementation was evaluated. Subsequently, the interaction between PpSKα and PpTrxh9 was determined. Lastly, the positive role of *PpSKα* in the enhancement of chilling tolerance through phosphorylation-dependent regulation of the expression of PpTrxh9 under GB treatment was elucidated.

## Materials and methods

### Plant materials and postharvest applications

Peach fruits (*Prunus persica* Batsch cv. Xiatian) at 80% maturation were collected from a plantation in Hefei, China. Uniformly sized peaches, with absence of physical injury or infection, were separated into two groups. Each group was randomly assigned to three small groups. A total of 190 peaches were used for each small group.

1. Control (CK): The fruits were soaked in distilled water.
2. GB group: The fruits were soaked in 10 mM GB solution.

After air-drying for approximately 1 h, all of the peaches were preserved at 4 ± 1°C with 80%–90% relative humidity for 35 days. For each small group, samples were taken at 7-day intervals. A total of 20 fruits were picked out to assess CI development and firmness. In addition, 10 fruits were picked out for the biochemical analysis, which were then stored at −80°C.

### Evaluation of CI severity and firmness

Cold-stored peaches were taken out of storage and then placed at 20°C for 3 days. The CI severity in each fruit was scored on the basis of the internal browning of peach fruits (Jia et al., 2023) and the external browning of tomato fruits (Acosta-Ramírez et al., 2024), as follows: 0, none; 1, <5%; 2, 6%–25%; 3, 26%–50%, and 4, >50%. The CI index was calculated as follows: CI index = Σ(CI scale × number of fruit within this scale)/(5 × total number of fruit).

Firmness was determined using a firmness detector. The diameters of the probe were set as 7.5 mm for peaches and 5.0 mm for tomatoes.

### Examination of the ROS content, MDA production, electrolyte leakage, and LOX and PLD activity

Liquid nitrogen-powdered fruit tissue was added to 10 mM Tris–HCl (pH 7.2) and then centrifuged at 11,000 × *g* at 4°C for 25 min. The obtained supernatant was diluted with 10 mM Tris–HCl (pH 7.2). Subsequently, the resulted samples were mixed with 2 mM 2′,7′-dichlorofluorescein diacetate. The reaction was carried out in the dark for 15 min. A fluorometer was used to determine the fluorescence values according to the method of Jambunathan (2010). The amount of ROS was described in arbitrary units per milligram fresh weight (FW).

Powdered fruit tissue was homogenized with 10% trichloroacetic acid. After centrifugation at 10,000 × *g* for 20 min, the obtained supernatant was added to 0.6% thiobarbituric acid in 10% trichloroacetic acid. The malondialdehyde (MDA) concentration was determined according to Zhang et al. (2013). The MDA content is presented in millimoles per kilogram FW.

A cork borer was used to obtain fruit disks with a thickness of 3 mm of equatorial mesocarp tissue. These disks were immersed in distilled water. Dried samples were added to 0.3 M mannitol. After 3 h, electrolyte leakage was assessed as reported by Zhang et al. (2013). The relative electrolyte leakage was calculated as a percentage of the total conductivity.

Powdered fruit samples were extracted in 0.1 M phosphate buffer (pH 6.8) and then subjected to centrifugation at 11,000 × *g* for 20 min at 4°C. The supernatant was used to assay the lipoxygenase (LOX) activity using the method of Liu et al. (2011). This measure is presented in units per gram FW.

Powdered fruit samples were added to 0.1 M acetic acid buffer (pH 5.6) and then centrifuged at 11,000 × *g* for 20 min at 4°C. The obtained supernatant was used to conduct assessment of the phospholipase D (PLD) activity based on the method of Liu et al. (2011). This measure is presented in units per gram FW.

### qRT-PCR analysis

A TRIzol kit (Tiangen Biotech, Beijing, China) was used to collect the total RNA. Subsequently, a PrimeScript™ RT Reagent Kit (Takara Bio Inc., Beijing, China) was used to obtain the first-strand cDNA. For quantitative real-time polymerase chain reaction (qRT-PCR) analysis, the primers for *PpTrxh9*, *PpSKα*, and *β-actin* in peach fruits are presented in **Supplementary Table S1**, while those for *SlTrxh9* and *β-actin* in tomato fruits are presented in **Supplementary Table S2**. The qRT-PCR procedures were based on Jia et al. (2022). The relative expression of the target genes was determined using the 2^−ΔΔCt^ method.

### Y2H test

The Matchmaker™ Gold Y2H System (Clontech, Mountain View, CA, USA) was utilized to perform the yeast two-hybrid (Y2H) assay. The coding sequences (CDSs) of *PpSKα* were fused with pGADT7 at the restriction sites *Sac*I and *BamH*I. The CDSs of *PpTrxh9* were fused with pGBKT7 at the restriction sites *BamH*I and *Pst*I. Yeast cells were co-introduced with different pairs of empty or reconstructed vectors. All of the co-transformants were cultured at 30°C on a two-deficiency selection medium (SD/−Leu−Trp) and a four-deficiency selection medium (SD/−Leu−Trp−Ade−His). Subsequently, the blue color was observed after X-α-galactosidase (Gal) was added to the four-deficiency selection medium. After 3 days, the yeast cultures co-transformed with different combinations of vectors were photographed.

### Determination of subcellular localization

The CDSs of *PpTrxh9* and *PpSKα* were inserted into pCAM35s–green fluorescent protein (GFP) at the restriction sites *Kpn*I and *Xba*I, respectively. The empty pCAM35s–GFP, pCAM35s–GFP–PpTrxh9, and pCAM35s–GFP–PpSKα vectors were electroporated into *Agrobacterium tumefaciens* GV3101. The suspensions of *A. tumefaciens* (OD_600_ = 0.6) containing the empty and reconstructed vectors were inoculated into the abaxial side of *Nicotiana benthamiana* leaves via 1-ml needleless syringes as reported in Stephan et al. (2011). mCherry was utilized to mark the cytoplasm localization. After infusion for 3 days, a confocal laser scanning microscope (Nikon A1-SHS, Tokyo, Japan) was used to observe the subcellular localizations of the target proteins.

### BiFC determination

The CDSs of *PpTrxh9* and *PpSKα* were separately ligated into the pSPYNE and pSPYCE vectors at the restriction sites *Xba*I and *BamH*I, respectively. *A. tumefaciens* EHA105 was introduced with the empty or the reconstructed vectors. The various pairs of vectors were inoculated into tobacco leaves. mCherry, which was used in the subcellular localization test, was also utilized in this experiment. After cultivation of the tobacco plants for 3 days, all images of the signals in infected leaves were taken using a confocal laser scanning microscope.

### Co-IP test

The CDSs of *PpTrxh9* were ligated into the pCAMBIA1300–35S–Myc vector at the restriction sites *BamH*I and *Sal*I. The CDSs of *PpSKα* were ligated into the pCAMBIA1300–35S–3×Flag vector at the restriction sites *BamH*I and *Sal*I. Tobacco leaves were injected with *A. tumefaciens* GV3101 harboring empty or recombinant plasmids. After 48 h, the total protein in liquid nitrogen-powdered tobacco leaves was obtained using a lysis buffer (50 mM Tris–HCl, pH 7.5, 150 mM NaCl, 5 mM DTT, 1 mM PMSF, 10 mM EDTA, 0.1% Triton X-100, and 1× protease inhibitor cocktail) and then incubated with anti-Flag magnetic beads for 12 h at 4°C. Western blot analysis was conducted to detect the target proteins using anti-Flag and anti-Myc antibodies.

### Pull-down test

The CDSs of *PpSKα* were inserted into the pGEX-4T-1 vector carrying the glutathione S-transferase (GST) tag at the restriction sites *EcoR*I and *BamH*I. The CDSs of *PpTrxh9* were inserted into the pET-28a vector carrying the His tag at the restriction sites *Xho*I and *BamH*I. GST, GST–PpSKα, and His–PpTrxh9 were introduced into the *Escherichia coli* strain BL21. After purification, His–PpTrxh9 was incubated with GST or with GST–PpSKα. The separation of proteins was carried out with 10% sodium dodecyl sulfate–polyacrylamide gel electrophoresis (SDS-PAGE). The target proteins were analyzed using Western blot detection with anti-GST and anti-His antibodies.

### Generation of tomato fruits overexpressing *PpSKα*

The CDSs of *PpSKα* were inserted into the pBI121 vector at the restriction sites *Xba*I and *Sma*I. *A. tumefaciens* GV3101 was introduced with the empty pBI121 and the resulting pBI121–*PpSKα* vectors thereafter. The generation of tomato plants (*Solanum lycopersicum* L. cv. MicroTom) overexpressing *PpSKα* was conducted via infection with *A. tumefaciens* transformants according to the method of Hao et al. (2015). Quantitative PCR (qPCR) assay was performed to measure the *PpSKα* expression in the wild-type (WT) and *PpSKα*-overexpressed (OE) plants (**Supplementary Figure S1**). The WT and the derived T3 generation lines were cultivated in a phytotron with a 16-h light (25°C)/8-h dark (20°C) cycle. Tomato fruits at the 80% plump stage were harvested. Subsequently, the 28-day cold storage and the collection of fruit samples proceeded as described in “*Plant materials and postharvest applications*.”

### Construction of peach fruits silencing *PpSKα*

The tobacco rattle virus (TRV)-based vector at the restriction sites *Kpn*I and *Xho*I was utilized to generate the virus-induced gene silencing (VIGS) construct of *PpSKα*. Subsequently, the empty and pTRV–*PpSKα* vectors were mobilized into *A. tumefaciens* GV3101. Suspensions of *A. tumefaciens* (OD_600_ = 1.0) carrying the empty and VIGS plasmids were utilized to infect peach fruits using a 1-ml syringe according to the method of Min et al. (2018). Refrigerated storage for 2 days and the subsequent sampling were performed as detailed in “*Plant materials and postharvest applications*.” An infection area with a diameter of 1.5 cm and a depth of 2.0 cm in peaches was sampled.

### Western blot test

The fruit samples were added into a radioimmunoprecipitation assay lysis buffer. Thereafter, the mixture was subjected to centrifugation at 11,000 × *g* for 25 min at 4°C. SDS-PAGE was used to separate the collected lysates. The subsequent Western blot analysis was performed based on the method of Jiao et al. (2016). The protein expression levels of PpTrxh9, PpSKα, and SlTrxh9 were normalized to that of RuBisCo.

### Data analysis

All datasets were subjected to one-way analysis of variance and Duncan’s multiple range test with SPSS 22.0 (SPSS Inc., Chicago, IL, USA). Significance of the differences among all treatments was measured at the 0.05 level.

## Results

### GB treatment improved the chilling tolerance and PpTrxh9 expression in peach fruits

**Figure 1:**
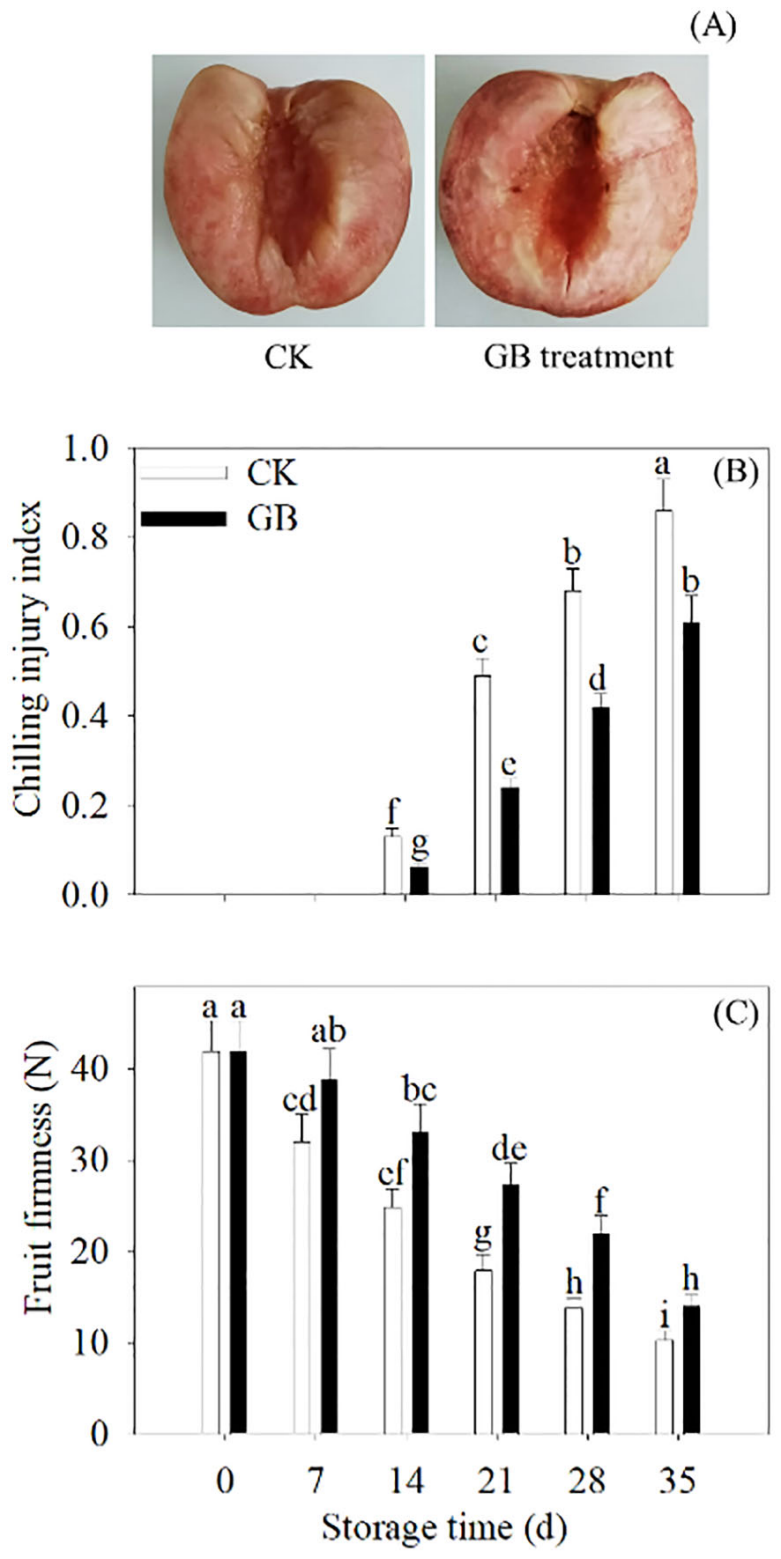
GB treatment potentiated the cold tolerance in peaches. The photographs of phenotypes of 35 days of cold-stored fruit **(A)** were taken. The CI index **(B)** and firmness **(C)** were detected every 7 days during 35 days of cold storage. The scale bars represent 1.5 cm. Each value represents average value ± standard deviation (SD). Different lowercase letters show significant differences at the 0.05 level according to Duncan tests.

CI symptoms in stored peach fruits became visible after 14 days. Compared with the control, cold-stored peach fruits supplemented with GB exhibited less internal browning. Moreover, GB-treated peaches showed lower CI index and higher firmness (**Figure 1**).

As demonstrated in the transcriptomic experiments and qRT-PCR, cold stress affected the gene expression of *PpTrxf*, *PpTrxh2*, *PpTrxh4-1*, *PpTrxh9*, *PpTrxm3*, *PpTrxo2*, *PpTrxx*, and *PpTrxy1*. Among the eight *PpTrxs*, the enhancement of the expression of *PpTrxh9* in response to cold treatment was the largest (**Supplementary Table S3**, **Supplementary Figure S2**). Therefore, PpTrxh9 was selected as the target protein for the subsequent determination of the molecular mechanisms of the GB-delayed CI progression.

**Figure 2:**
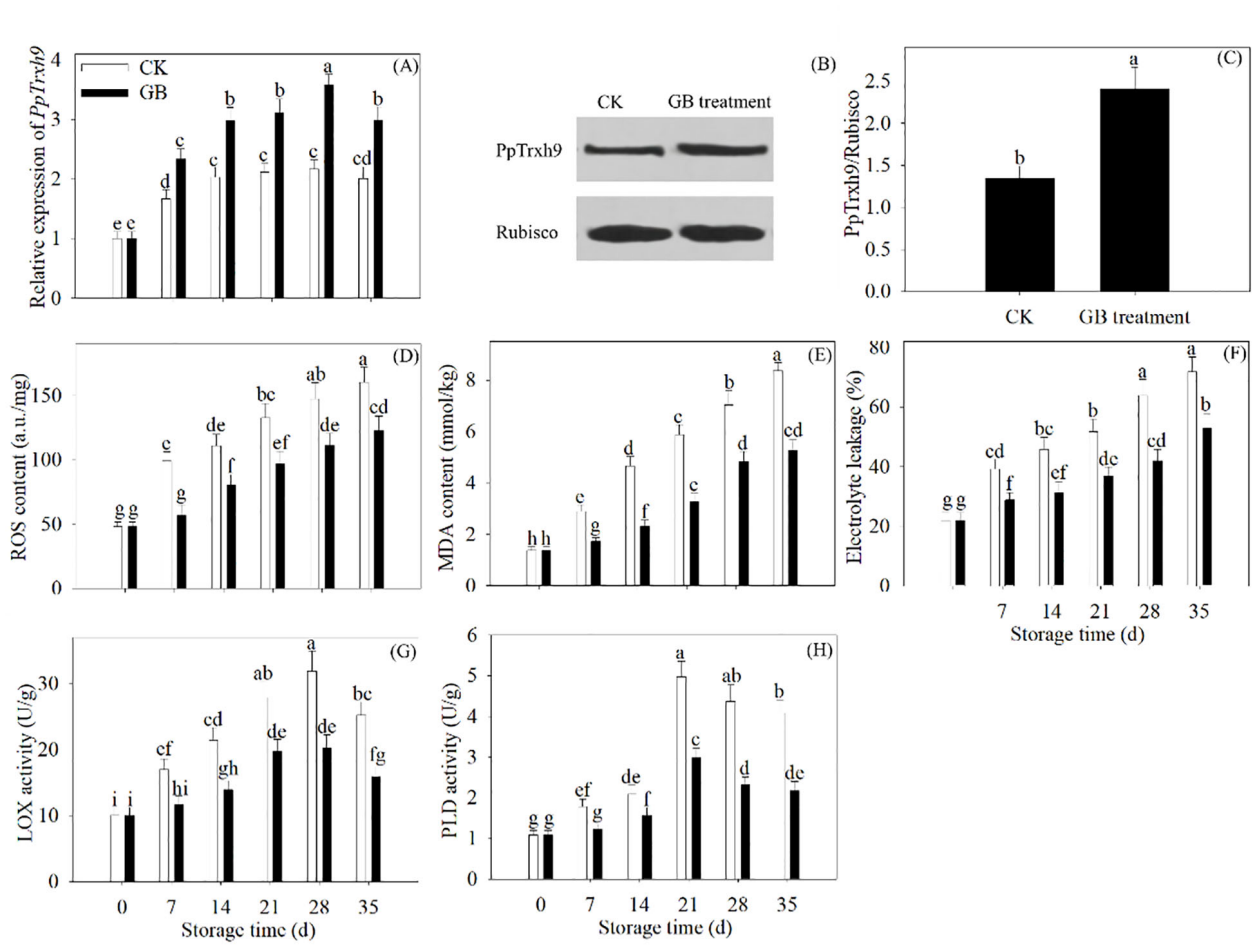
GB treatment improved the PpTrxh9 expression and inhibited the oxidative damage in peaches. **(A)** The gene expression of *PpTrxh9* was detected every 7 days during 35 days of refrigerated preservation. **(B, C)** The protein expression of PpTrxh9 was evaluated in 35 days of cold-stored peaches. **(D-H)** The ROS production, MDA generation, electrolyte leakage and activity of LOX and PLD were assayed every 7 days during 35 days of cold storage. Each value represents average value ± standard deviation (SD). Different lowercase letters show significant differences at the 0.05 level according to Duncan tests.

The gene expression of *PpTrxh9* was enhanced by GB supplementation throughout the refrigeration process. After 35 days of refrigerated storage, GB treatment improved the protein expression of PpTrxh9. Furthermore, GB treatment decreased the ROS generation. The MDA content, the electrolyte leakage, and the LOX and PLD activity also decreased with GB treatment (**Figure 2**).

### The PpSKα protein interacted with the PpTrxh9 protein

**Figure 3:**
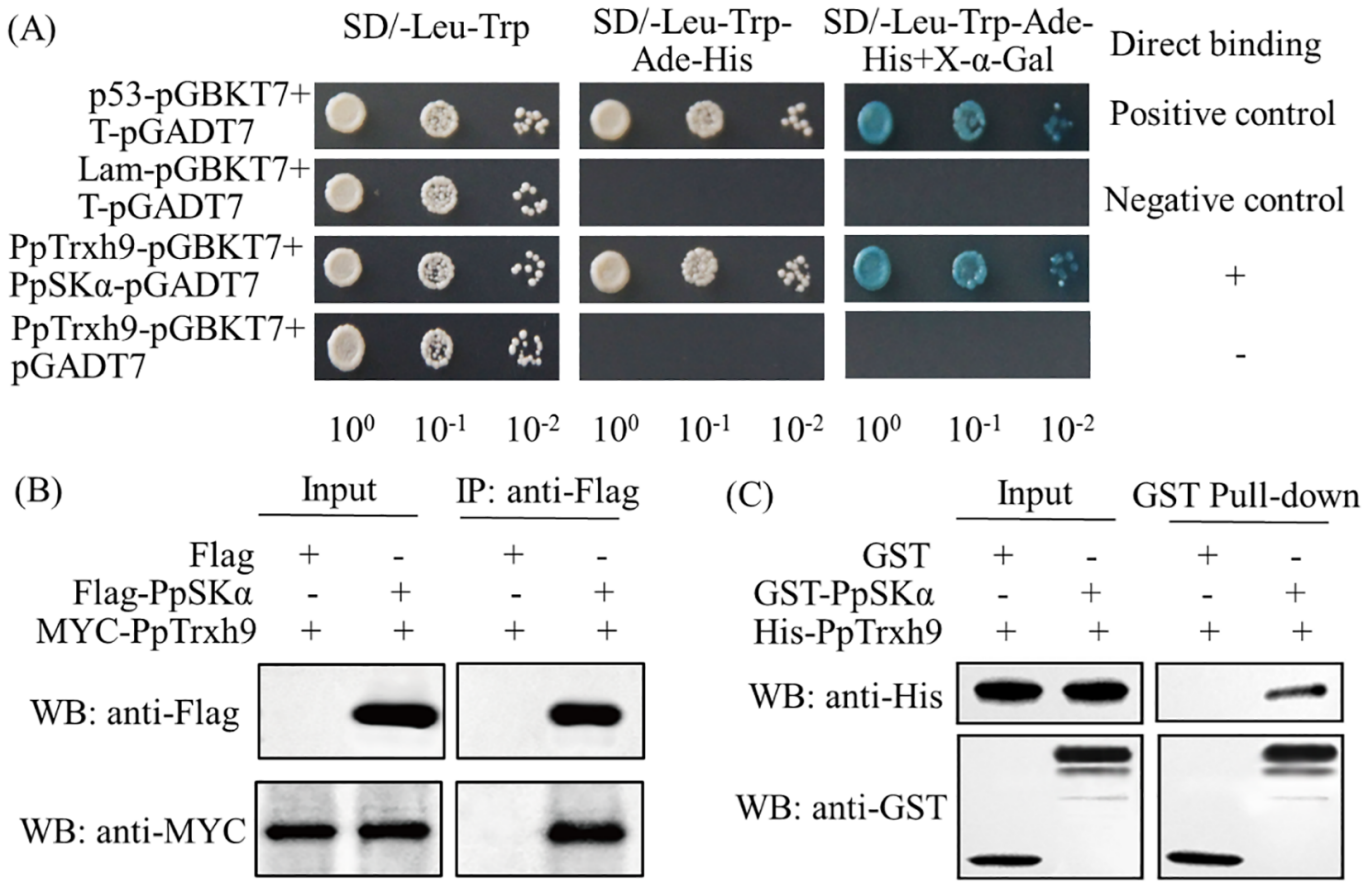
Assays of Y2H, Co-IP and pull-down showed that PpSKα protein interacted with PpTrxh9 protein. **(A)** Y2H test. The yeast colonies cultivated for 3 days at 28°C were observed. The positive interaction was indicated by the normal growth of yeast cells cultured on SD/-Leu-Trp-Ade-His medium and the generation of blue yeast colonies after X-α-Gal was added. **(B)** Co-IP detection. Flag-PpSKα or empty Flag (negative control) and MYC-PpTrxh9 were co-expressed in tobacco leaves. Total proteins in the co-expressed leaves were immunoprecipitated with anti-Flag antibody. **(C)** Pull-down assay. His-PpTrxh9 protein was incubated with GST-PpSKα or empty GST (negative control). Immunoblot tests were used to detect the bound proteins with anti-His and anti-GST antibodies.

As seen from the Y2H screening, PpSKα was identified as a possible protein interacting with PpTrxh9. The Y2H assay was performed to verify the interaction between PpSKα and PpTrxh9. The transformants harboring pGBKT7–PpTrxh9 and pGADT7–PpSKα grew well on the SD/−Leu−Trp−Ade−His medium and turned blue in the presence of X-α-Gal. The transformants harboring pGBKT7–PpTrxh9 and the empty pGADT7 vector did not grow well on the SD/−Leu−Trp−Ade−His medium. These results suggest the interaction of PpSKα with PpTrxh9 (**Figure 3A**).

In the co-immunoprecipitation (Co-IP) assay, following immunoprecipitation with the anti-Flag antibody, MYC–PpTrxh9 co-precipitated with Flag–PpSKα, but not with the empty Flag. In addition, in the pull-down assay, GST–PpSKα could pull down His–PpTrxh9, while the empty GST could not. These two experiments confirmed the interaction between PpSKα and PpTrxh9 (**Figures 3B, C**).

**Figure 4:**
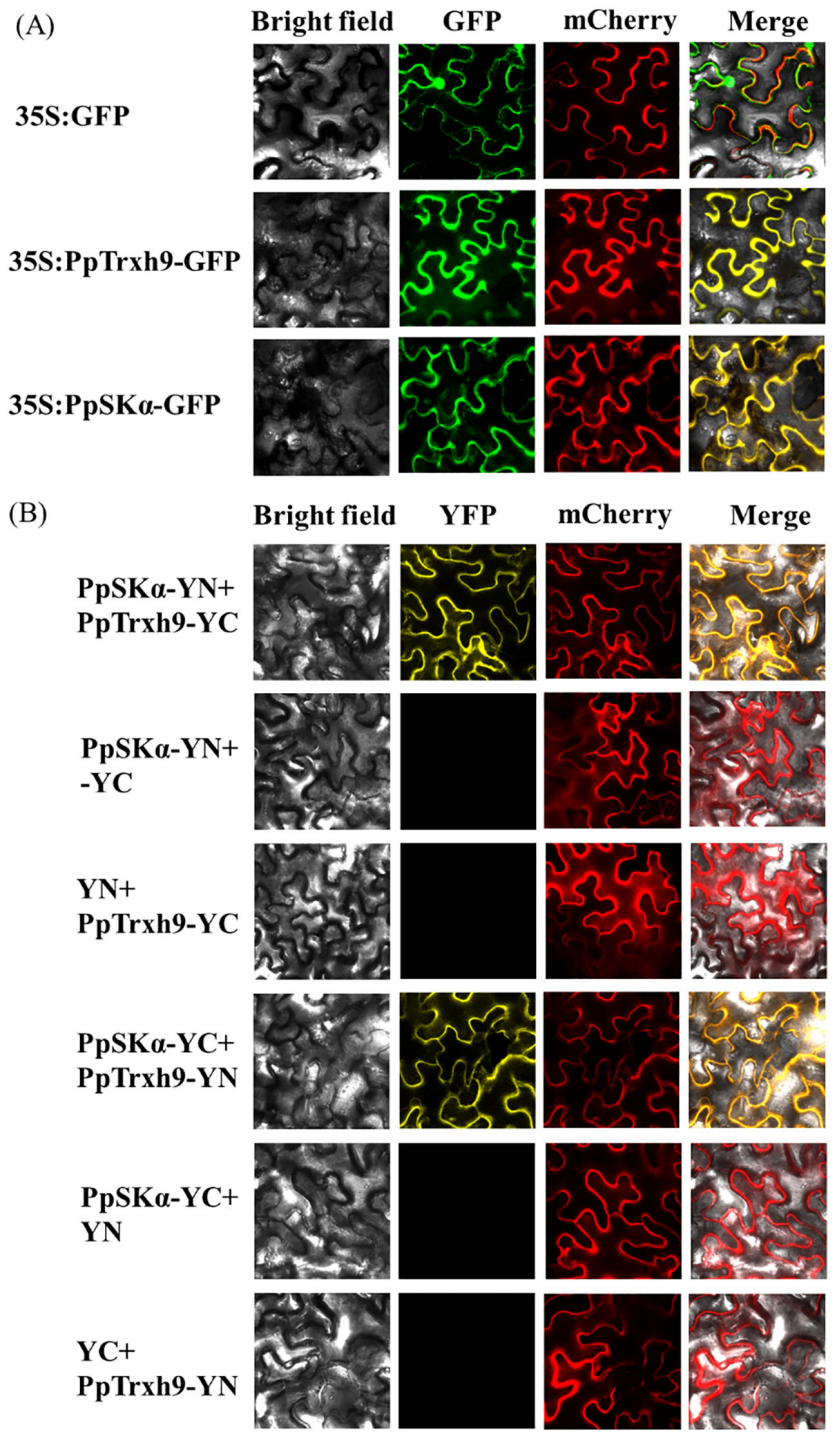
BiFC experiment furtherly verified the protein-protein interaction between PpSKα and PpTrxh9. **(A)** Subcellular localization of PpTrxh9 and PpSKα. Bars=30 μm. The empty GFP, GFP-PpTrxh9 and GFP-PpSKα vectors were transferred into tobacco leaves. mCherry served to locate the cytoplasm. Confocal laser scanning microscope was used to visualize the expression of GFP and mCherry. **(B)** BiFC test validated that the interaction between PpSKα and PpTrxh9 occurred in the cell membrane and cytoplasm. Bars=30 μm. mCherry was applied as the cytoplasm localization marker. The presence of YFP fluorescent signals represented the positive interaction.

Furthermore, as suggested in the subcellular localization assay, both PpSKα and PpTrxh9 were localized in the cell membrane and the cytoplasm. Subsequently, bimolecular fluorescence complementation (BiFC) detection was conducted to further validate the interaction. Yellow fluorescent protein (YFP) fluorescence signals were observed in the membrane and the cytoplasm in tobacco cells co-expressing PpSKα-YN plus PpTrxh9-YC and PpSKα-YC plus PpTrxh9-YN. YFP fluorescence signals were not observed when PpSKα-YN plus YC, YN plus PpTrxh9-YC, PpSKα-YC plus YN, and YC plus PpTrxh9-YN were co-transformed. Taken together, the findings corroborated the interaction of PpSKα with PpTrxh9 in the cell membrane and the cytoplasm (**Figure 4**).

### GB treatment boosted PpSKα expression

**Figure 5:**
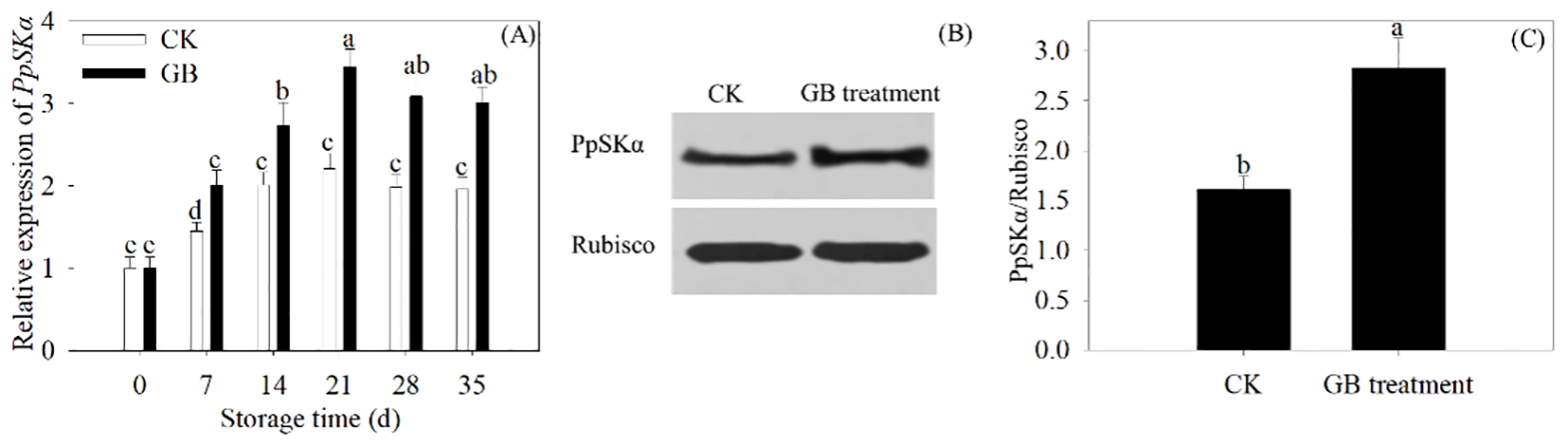
GB application boosted the PpSKα expression in peach fruit. **(A)** The gene expression of *PpSKα* was assayed every 7 days during 35 days of cold storage. **(B, C)** The protein expression of PpSKα in 35 days of cold-stored peaches. Each value represents average value ± standard deviation (SD). Different lowercase letters show significant differences at the 0.05 level according to Duncan tests.

During 35 days of refrigerated storage, the gene expression of *PpSKα* increased with GB supplementation in peach fruits. GB treatment promoted the protein expression of PpSKα in 35-day cold-stored peaches (**Figure 5**). The results displayed the enhancement of the gene expression of *PpSKα* with GB supplementation.

### Overexpression of *PpSKα* in tomato fruits and VIGS of *PpSKα* in peach fruits altered the cold resistance

**Figure 6:**
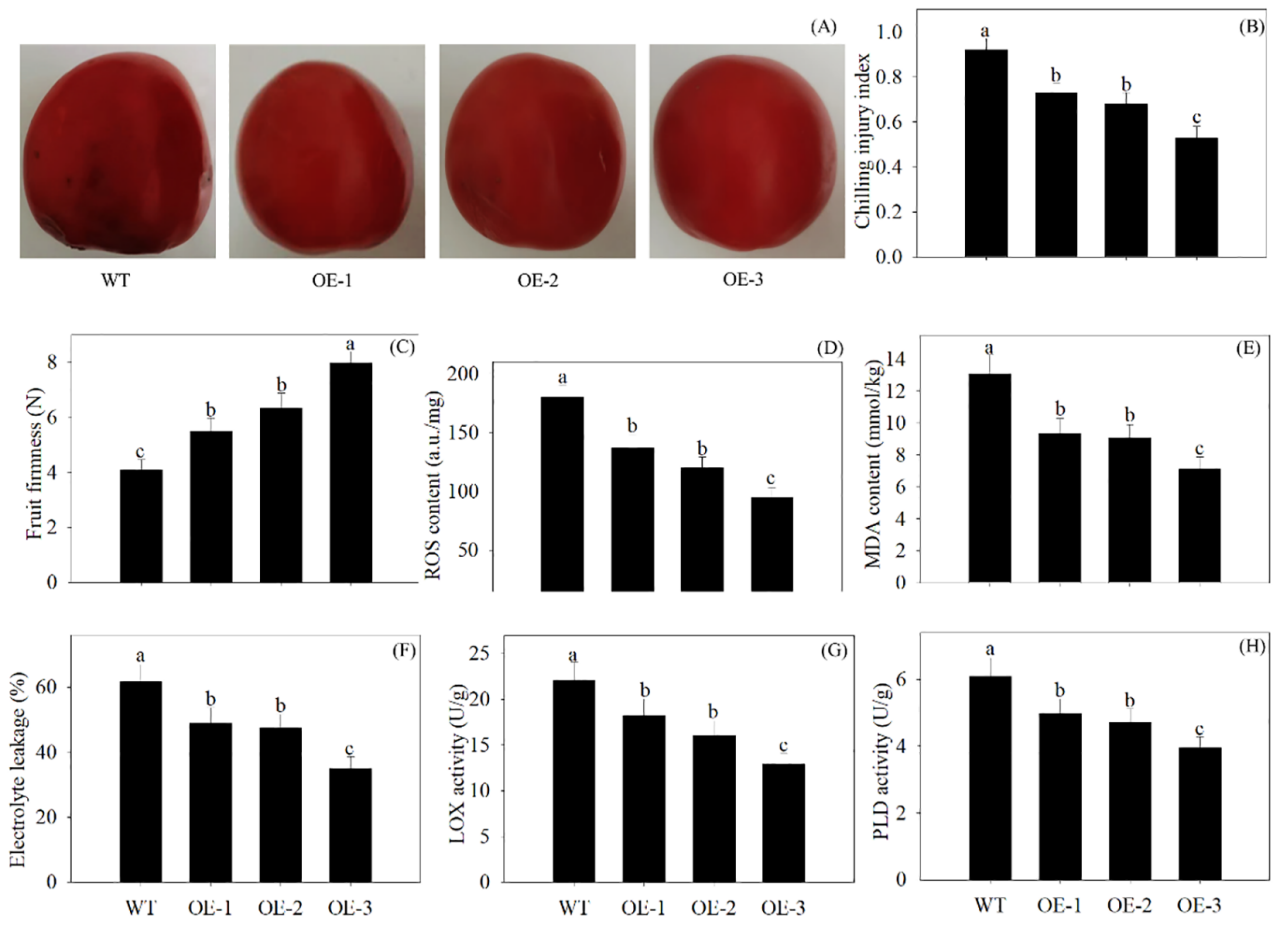
Overexpression of *PpSKα* in tomato fruit lowered the cold sensitivity. The phenotypes **(A)** were observed, and the CI development **(B)**, firmness **(C)**, ROS generation **(D)**, MDA production **(E)**, electrolyte leakage **(F)** and activity of LOX **(G)** and PLD **(H)** were assayed in 28 days of cold-stored tomatoes. The scale bars represent 1 cm. Each value represents average value ± standard deviation (SD). Different lowercase letters show significant differences at the 0.05 level according to Duncan tests.

In comparison with the WT, the overexpression of *PpSKα* inhibited the visible external browning in 28-day cold-stored tomato fruits. Tomato fruits overexpressing *PpSKα* exhibited lower CI progression and higher firmness. The ROS production, MDA content, electrolyte leakage, and the activity of LOX and PLD decreased in the *PpSKα*-OE tomato fruits (**Figure 6**). The findings illustrated that *PpSKα* lowered the CI degree in tomatoes.

**Figure 7:**
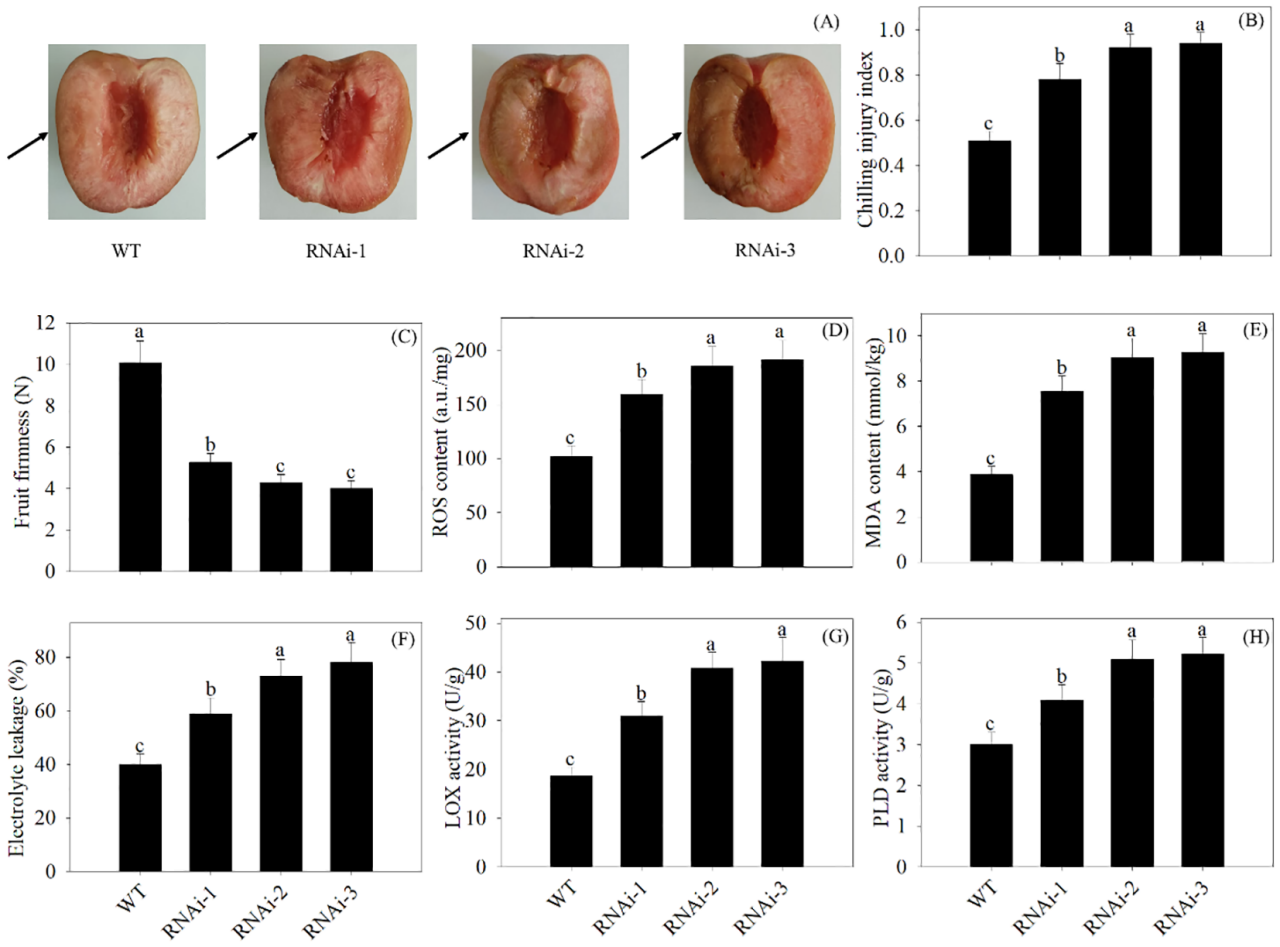
VIGS of *PpSKα* in peach fruit weakened the chilling tolerance. The photographs of phenotypes **(A)** were taken, and the CI progression **(B)**, firmness **(C)**, ROS accumulation **(D)**, MDA content **(E)**, electrolyte leakage **(F)** and activity of LOX **(G)** and PLD **(H)** were evaluated in 2 days of cold-stored peaches. The scale bars represent 1.5 cm. Each value represents average value ± standard deviation (SD). Different lowercase letters show significant differences at the 0.05 level according to Duncan tests.

In comparison with the WT, VIGS of *PpSKα* resulted in more internal browning in 2-day cold-stored peach fruits. The *PpSKα-*silenced peaches showed higher CI and lower firmness. Furthermore, VIGS of *PpSKα* led to increases in the ROS production, MDA content, electrolyte leakage, and LOX and PLD activity (**Figure 7**). The results elucidated the positive impact of *PpSKα* on boosting the cold resistance in postharvest peach fruits.

### PpSKα phosphorylated PpTrxh9

**Figure 8:**
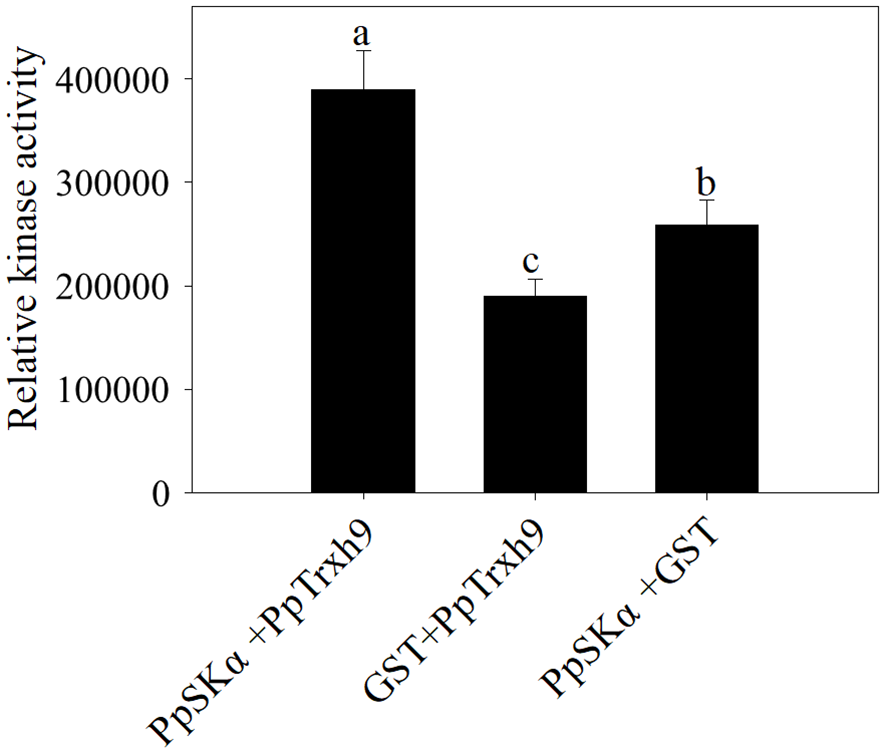
The phosphorylation of PpTrxh9 by PpSKα. The kinase reactions were conducted *in vitro*. GST was applied to be the negative control. Each value represents average value ± standard deviation (SD). Different lowercase letters show significant differences at the 0.05 level according to Duncan tests.

A fluorometric kinase test was conducted to determine the phosphorylation of PpTrxh9 by PpSKα. After incubation of PpSKα with GST, the relative kinase activity was detectable, which showed auto-phosphorylation of PpSKα. Compared with the control (following the incubation of PpTrxh9 with GST), the relative kinase activity was higher following incubation of PpSKα with PpTrxh9. These data suggest that PpSKα phosphorylated PpTrxh9 (**Figure 8**).

### Overexpression of *PpSKα* in tomato fruits and VIGS of *PpSKα* in peach fruits affected the Trxh9 expression

**Figure 9:**
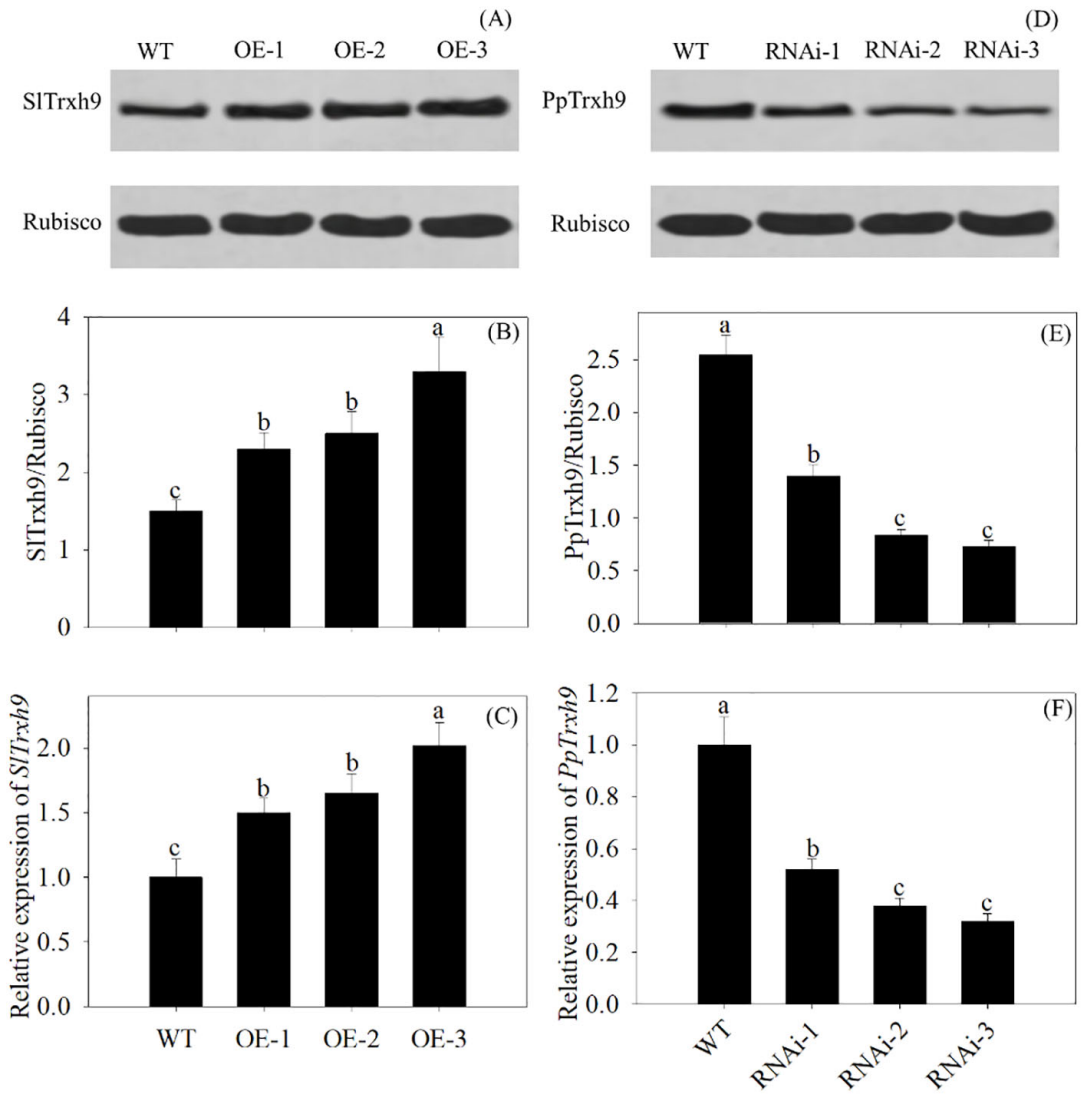
The Trxh9 expression in the *PpSKα*-OE tomato fruit and *PpSKα*-silenced peach fruit. **(A-C)** The expression of protein and gene of SlTrxh9 was assayed in 28 days of cold-stored tomato fruit. **(D-F)** The expression of protein and gene of PpTrxh9 in 2 days of cold-stored peach fruit. Each value represents average value ± standard deviation (SD). Different lowercase letters show significant differences at the 0.05 level according to Duncan tests.

Compared with the WT, the overexpression of *PpSKα* upregulated the protein and gene expression of SlTrxh9 in 28-day cold-stored tomato fruits, whereas VIGS of *PpSKα* downregulated the protein and gene expression of PpTrxh9 in 2-day cold-stored peach fruits. This phenomenon illustrated the contribution of *PpSKα* to the increase in PpTrxh9 expression in peach fruits (**Figure 9**).

## Discussion

GB supplementation decreased the sensitivity to cold stress in peaches (**Figure 1**). The disruption of ROS homeostasis under cold stress gives rise to oxidative damage, which is responsible for CI development in postharvest fruits (Jiao and Sun, 2024; He et al., 2025). Many antioxidant substances play essential functions in suppressing ROS overproduction in plants. The Trxs, as small ubiquitous proteins, play critical roles in the defense against oxidative stress, influencing the plant responses to stressful conditions (Geigenberger et al., 2017). The addition of GB notably upregulated the gene and protein expression of PpTrxh9 (**Figures 2A–C**). Subsequently, decreases in the ROS production, MDA content, electrolyte leakage, and LOX and PLD activity with GB treatment were observed (**Figures 2B–F**). Excessive ROS in prolonged cold-stored fruits would cause destruction of the cell membrane. The decreases in the MDA content, electrolyte leakage, and LOX and PLD activity retarded the membrane lipid peroxidation and maintained the membrane integrity (Wang et al., 2022). Accordingly, tobacco plants overexpressing *SlTrxh* from tomatoes showed a decrease in the accumulation of MDA and ROS and an enhancement of the resistance to excess nitrate stress (Zhai et al., 2022). *OsTRXh1* from rice facilitated the reduction in ROS production, thus altering the susceptibility to salt and abscisic acid stresses (Zhang et al., 2011). It could be indicated that the function of plant Trxs in ROS removal is crucial for enhancing the tolerance to multiple stresses. Thus, it was concluded that GB supplementation decreased the oxidative injury by activating the expression of *PpTrxh9*, facilitating to render the peach fruits more resistant to cold stress. The regulation of redox balance by manipulating this target gene could potentiate the defense responses to low-temperature stress following exogenous applications during prolonged cold storage in postharvest fruits.

As shown in the Y2H, Co-IP, pull-down, and BiFC assays, a GSK3/SK, i.e., PpSKα, was capable of interacting with PpTrxh9 in the cell membrane and the cytoplasm (**Figures 3**, **4**). GSK3/SKs, as a family of serine/threonine protein kinases, act as key players in a series of biological processes (Zolkiewicz and Gruszka, 2022). GB-treated peach fruits showed an increase in the expression of the gene and protein of PpSKα (**Figure 5**). Based on the phenomenon in our previous reports that the use of bikinin (GSK3/SK inhibitor) resulted in the repression of cold resistance in peach fruits (Jiao and Duan, 2019a, 2019), in this research, the molecular mechanisms underlying the regulation of cold resistance by *PpSKα* were unraveled. The overexpression of *PpSKα* in tomatoes decreased the sensitivity to cold stress, whereas VIGS of *PpSKα* in peaches caused the aggravation of CI progression (**Figures 6A–C**, **7A–C**). These findings exactly substantiated that *PpSKα* contributed to the suppression of CI development in postharvest peaches. Accordingly, *SK12* in *Arabidopsis* negatively regulated the resistance to cold stress (Guan et al., 2013). The BIN2 (GSK3-like kinase) in *Arabidopsis* was also found to lower the cold tolerance (Li et al., 2017; Ye et al., 2019). These findings elucidate the negative roles of GSK3/SKs in chilling resistance in *Arabidopsis*. Knockout mutation of the rice *OsGSK1* conferred enhanced tolerance to cold stress, suggesting the negative function of *OsGSK1* in chilling resistance in rice (Koh et al., 2007). Collectively, our results and previous reports demonstrated that the different members of GSK3/SKs in different species exert opposite effects on the defense against low-temperature stress. Our research shed light on the positive function of *PpSKα* in repressing CI during postharvest preservation. Further data illustrated that the oxidative damage was attenuated in *PpSKα*-OE tomato fruits, but was aggravated in *PpSKα*-silenced peach fruits (**Figures 6D–H**, **7D–H**). Thus, *PpSKα* was conducive to lowering the oxidative injury, positively regulating the chilling tolerance in cold-stored peaches. In parallel, BIN2 (Lu et al., 2022) and ASKα (Dal Santo et al., 2012) from *Arabidopsis*, *GmBIN2* from soybean (Wang et al., 2018), and *MmSK* from mulberry (Li et al., 2018) have been reported to repress oxidative damage, thus enabling plants to adapt to stress conditions.

Lastly, the results of the kinase assay suggest that PpSKα phosphorylated PpTrxh9 (**Figure 8**). Phosphorylation of target proteins by serine/threonine protein kinases, as one of the posttranslational modifications, affects diverse downstream biological events. Further analysis showed that tomato fruits overexpressing *PpSKα* displayed elevation of SlTrxh9 expression, whereas peach fruits silencing *PpSKα* showed reduction of PpTrxh9 expression (**Figure 9**). Taken together, our results elucidated that *PpSKα* relieved the oxidative damage through interaction with and the phosphorylation-dependent regulation of PpTrxh9, thus retarding the CI progression in peaches. Similarly, the interaction between BIN2 and ICE1 from *Arabidopsis* was demonstrated, and BIN2 was found to phosphorylate and destabilize ICE1, thereby compromising the cold resistance (Ye et al., 2019). It appears that the phosphorylation reactions of target genes by GSK3/SKs are crucial regulatory events in response to cold stress.

**Figure 10:**
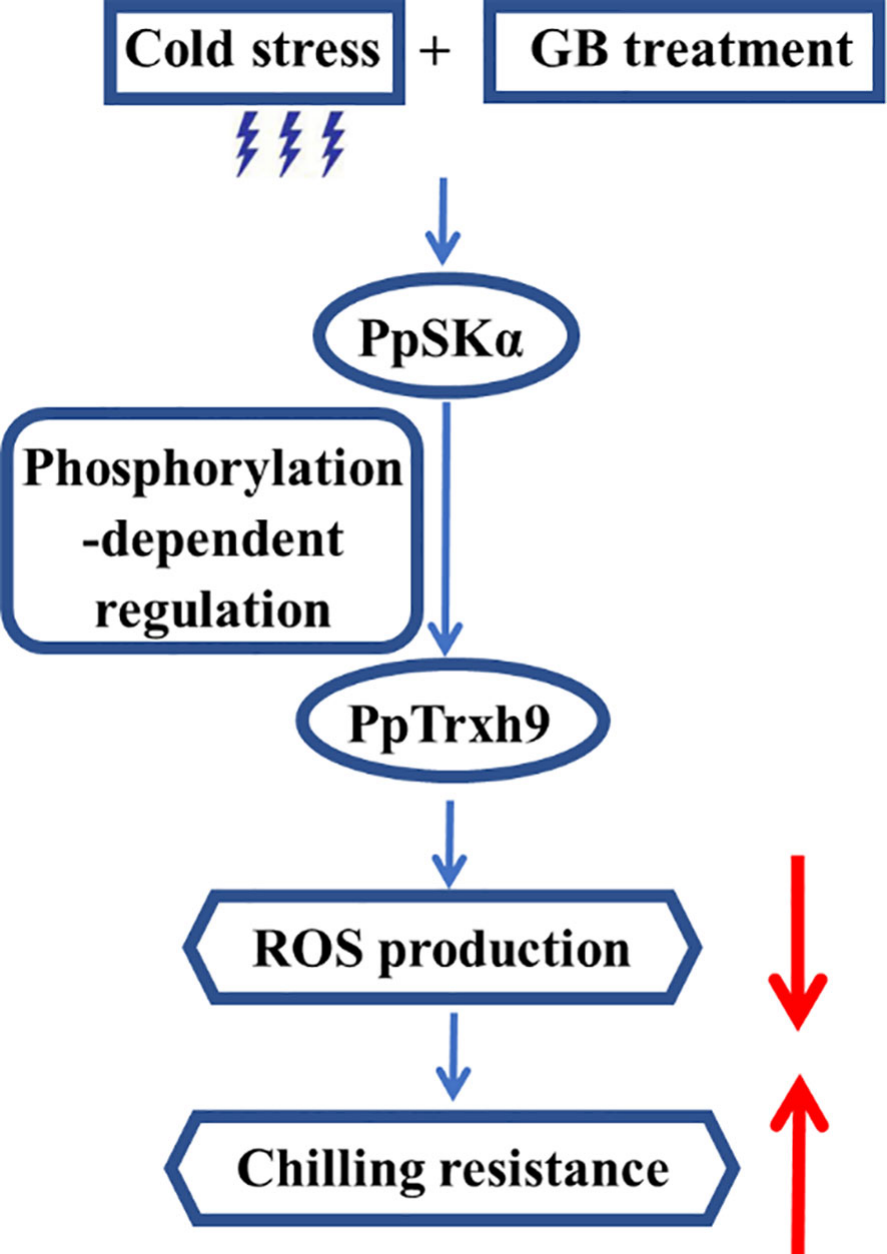
A proposed working model of the involvement of PpSKα in the GB application-boosted chilling resistance by enhancing PpTrxh9 expression in peaches.

According to all of the above results, it could be presumed that GB treatment triggered the PpSKα expression under cold stress. The interaction between PpSKα and PpTrxh9 occurred. PpSKα boosted the PpTrxh9 expression via phosphorylation reaction. The PpSKα–PpTrxh9 pathway contributed to the defense against oxidative stress, thereby diminishing the susceptibility of peaches to cold stress (**Figure 10**). The results of the present study open up an avenue for genetic maintenance of quality in postharvest fruits.

## Conclusion

GB treatment ameliorated the CI in peaches. GB-treated peaches showed higher PpTrxh9 expression and less oxidative injury. The results of the Y2H, Co-IP, pull-down, and BiFC assays revealed that PpSKα interacted with PpTrxh9 in the cell membrane and the cytoplasm. PpSKα expression was found to be enhanced by GB supplementation in peach fruits. The cold sensitivity and oxidative damage decreased in *PpSKα-*OE tomato fruits, but increased in *PpSKα-*silenced peach fruits. The phosphorylation of PpTrxh9 by PpSKα was indicated by a kinase assay. Moreover, the overexpression of *PpSKα* in tomato fruits boosted the expression of SlTrxh9, whereas VIGS of *PpSKα* in peach fruits reduced the expression of PpTrxh9. Taken together, PpSKα promoted the expression of PpTrxh9 and thus compromised the CI in peach fruits.

## Data Availability

The original contributions presented in the study are included in the article/**Supplementary Material**. Further inquiries can be directed to the corresponding author.
