# Supplementary material for: PpSKα boosts chilling tolerance by activating PpTrxh9 in peach fruit
Source: Front Plant Sci. 2025 Jun 13;16:1603423. doi: 10.3389/fpls.2025.1603423 (PMC12202609; doi:10.3389/fpls.2025.1603423)
Supplement: Supplementary file 1 [file DataSheet1.doc]

**Pp****SKα boosts chilling tolerance by activating** **PpTrxh9 in peach fruit**

Caifeng Jiao ^a^ *, Jing Sun ^b^

^a^ School of Horticulture, Anhui Agricultural University, Hefei 230036, People’s Republic of China

^b^ College of Food Science and Engineering, Nanjing University of Finance and Economics,

Nanjing 210023, People’s Republic of China

***Corresponding Author**

Email address: anyeweiyangjiao@126.com

Tel/Fax: 86-0551-65786441

**Figure S1.** Relative expression of *PpSKα* in WT and T3 transgenic tomato plants. Each value represents average value ± standard deviation (SD). Different lowercase letters show significant differences at the 0.05 level according to Duncan tests.

**
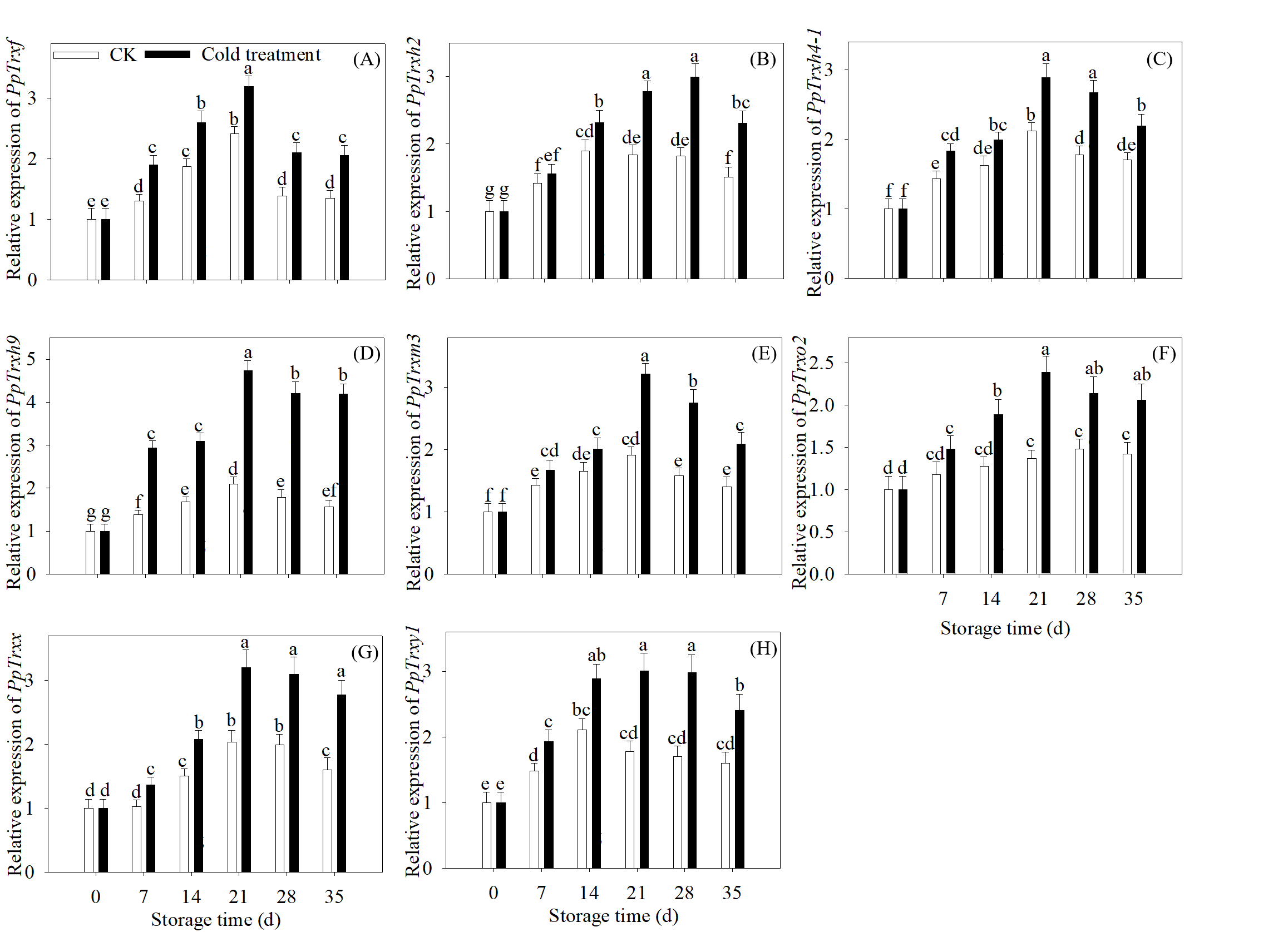
**

**Figure S2.** The induction of gene expression of *PpTrxs* by cold storage in peach fruit. Each value represents average value ± SD. Different lowercase letters show significant differences at the 0.05 level according to Duncan tests.

**Table S1.** The primers for qRT-PCR detection in peach fruit.

| Gene | Accession number | Primer name | Primer sequences (5’→3’) |
| --- | --- | --- | --- |
| *PpTrxh9* | XM_020567179.1 | Sense | TCTCATATTGCACGCACTCG |
|  |  | Antisense | CCCTGAAAGCTTTAGAGTACAATC |
| *PpSKα* | XM_007205208.2 | Sense | ACAGGTTTGCAACGAATGGC |
|  |  | Antisense | TGTTGGAGTGCCCAAAACCT |
| *β-actin* | XM_007211382.2 | Sense | GTTATTCTTCATCGGCGTCTTCG |
|  |  | Antisense | CTTCACCATTCCAGTTCCATTGTC |

**Table S2.** The primers for qRT-PCR detection in tomato fruit.

| Gene | Accession number | Primer name | Primer sequences (5’→3’) |
| --- | --- | --- | --- |
| *SlTrxh9* | XM_004246235.4 | Sense | TCTCAAGGATGGGGAGCAGA |
|  |  | Antisense | TTGCAATTACTGTGGCTGCG |
| *β-actin* | AB695290.1 | Sense | GAGCACGGAATTGTCAGCAA |
|  |  | Antisense | AGGGGCTTCAGTTAGGAGGA |

**Table S3. The differential expression of identified *PpTrxs* in cold-stored peaches through transcriptomic analysis.**

| Gene ID (LOC) | Gene Description | Log_2_^Fold change^ |
| --- | --- | --- |
| 18772104 | *Trxf* | 1.98±0.16 |
| 18781990 | *Trxh2* | 1.91±0.15 |
| 18786215 | *Trxh4-1* | 1.53±0.11 |
| 18775283 | *Trxh9* | 2.97±0.19 |
| 18767687 | *Trxm3* | 1.88±0.18 |
| 18780197 | *Trxo2* | 1.69±0.17 |
| 18792961 | *Trxx* | 2.21±0.21 |
| 18779870 | *Trxy1* | 1.90±0.18 |

|log_2_^Fold change^|≥1 represents up-regulation, while 0＜|log_2_^Fold change^|＜1 represents no statistical difference. Each value represents average value ± SD.
